# Novel subpopulations in date palm (*Phoenix dactylifera*) identified by population-wide organellar genome sequencing

**DOI:** 10.1186/s12864-019-5834-7

**Published:** 2019-06-17

**Authors:** Yasmin A. Mohamoud, Lisa S. Mathew, Maria F. Torres, Shameem Younuskunju, Robert Krueger, Karsten Suhre, Joel A. Malek

**Affiliations:** 1 Genomics Laboratory, Weill Cornell Medicine-Qatar, Doha, 24144 Qatar; 2 Department of Genetic Medicine, Weill Cornell Medicine-Qatar, Doha, 24144 Qatar; 3 Dipartimento di Scienze Agrarie e Forestali, Università degli Studi di Palermo, 90128 Palermo, Italy; 4 USDA-ARS National Clonal Germplasm Repository for Citrus & Dates, Riverside, CA USA; 5 Department of Physiology, Weill Cornell Medicine-Qatar, Doha, 24144 Qatar; 6 Present Address: Department of Biological Sciences, University of Cincinnati, Cincinnati, OH USA

**Keywords:** Date palm, Domestication, Cultivation, Organellar genome sequencing

## Abstract

**Background:**

The date palm is one of the oldest cultivated fruit trees. The tree can withstand high temperatures and low water and the fruit can be stored dry offering nutrition across the year. The first region of cultivation is believed to be near modern day Iraq, however, where and if the date palm was domesticated is still a topic of debate. Recent studies of chloroplast and genomic DNA revealed two major subpopulations of cultivars centered in both the Eastern range of date palm cultivation including Arabian Peninsula, Iraq and parts of South Asia, and the Western range, including North Africa.

**Results:**

To better understand the origins of date palm cultivation we sequenced and analyzed over 200 mitochondrial and chloroplast genomes from a geographically diverse set of date palms. Here we show that, based on mitochondrial and chloroplast genome-wide genotyping data, the most common cultivated date palms contain 4 haplotypes that appear associated with geographical region of cultivar origin.

**Conclusions:**

These data suggest at least 3 and possibly 4 original maternal contributions to the current date palm population and doubles the original number. One new haplotype was found mainly in Tunisia, Algeria and Egypt and the second in Iraq, Iran and Oman. We propose that earliest date palm cultivation occurred independently in at least 3 distinct locations. This discovery will further inform understanding of the history and origins of cultivated date palm.

**Electronic supplementary material:**

The online version of this article (10.1186/s12864-019-5834-7) contains supplementary material, which is available to authorized users.

## Background

The importance of date palm to early civilizations is well documented and it is among the earliest cultivated fruit trees [1]. There are hundreds of commercially important date palm cultivars across the main growing regions of North Africa, the Middle East, Arabian Gulf and western parts of South Asia. Despite its historical importance, little is known about its earliest development and whether it was truly domesticated or simply cultivated. This is complicated by the fact that highly favored cultivars of date palm are clonally propagated and likely have been since antiquity [2]. The absence of widely distributed wild date palm progenitors has further complicated the analysis though a recent study has identified potential wild date palms in Oman [3]. The date palm is dioecious with separate male and female trees and hybridization with other Phoenix species is possible and hybridization was recently shown to have likely occurred with *P. theophrasti* during the spread of date palm cultivation in North Africa [4].

Multiple studies, including our own, of Y chromosome or chloroplast markers and genome-wide SNP analysis has confirmed at least two major sub-populations in the date palm [5–9]. The subpopulations show strong distinction between North African (Western) and Arabian Gulf (Eastern) cultivars while admixture is observed in cultivars from Egypt, Sudan and the Middle East. High genetic diversity in the North Africa subpopulation argues against it simply a result of colonization from a middle-east population [8] though studies have shown a significant portion of the North Africa date palm genome likely originates from the Arabian Gulf date palm [3, 4, 10]. Despite these results, many have suggested that the date palm was originally domesticated in the region of modern day Iraq as the historical record of date palm is richest in that region [11]. Date palms do not figure in Egyptian hieroglyphics until the 12 century BC [12]. However, it has been argued that the date palm may have simply been a tree of horticultural importance in North Africa as opposed to its religious importance in the East where the historical record is documented earlier. Within this debate, others have suggested that the date palm was cultivated in multiple locations at different times and no single origin of cultivation will be located [13].

While analysis of nuclear genomic markers is highly informative for understanding genetic admixture patterns, little work has been done to study variation in organellar genomes of date palm cultivars from multiple geographical regions. Organellar genomes in angiosperms offer the benefit that, as in animals, they are transmitted from the maternal lineage with evidence for bipaternal transmission only in rare cases [14]. For these reasons, maternal transmission of organellar genomes could be of interest for studying the origins of date palm cultivation. Many groups have sequenced portions of the chloroplast genome and found numerous haplotypes within Tunisian [15, 16], Saudi Arabian [17] or Emirati cultivars [18]. This study presents organellar genome sequencing results from across these regions of cultivation.

## Results

We collected 201 date palms from across the main regions of cultivation (Table 1) and included *Brahea dulcis* and 4 *Phoenix* species for comparison (Additional file 1). Maximum sequencing coverage approached up to thousands of fold coverage, however, SNP calling was conducted on a maximum of 250 randomly selected reads per position. Average utilized sequence coverage for the samples was 223X and no sample had less than an average of 86X coverage across the two organelle genomes. We believe this is the first reported organellar genome sequencing from dried date palm fruit.

**Table 1:** Counts of samples from each country and distribution among the Mitochondrial haplotypes

Country	AG1	AG2	NA1	NA2	total
Algeria	12	0	13	3	28
Egypt	4	0	6	1	11
Iran	1	3	0	0	4
Iraq	3	0	0	0	3
Jordan	4	0	0	1	5
Saudi Arabia	17	3	1	0	21
Libya	1	0	4	0	5
Morocco	8	0	24	1	33
Oman	4	3	0	0	7
Pakistan	2	0	3	0	5
Qatar	5	2	0	0	7
Sudan	0	0	6	0	6
Tunisia	0	0	3	2	5
UAE	17	4	0	0	21
USA	12	2	15	10	40

Haplotypes labels are as in Fig. 2. UAE – United Arab Emirates

SNP filters that required at least one high quality alternative allele in any of the samples studied resulted in 177 SNPs identified in the 158,462 bp Chloroplast genome and 841 in the 715,001 bp Mitochondria genomes for a total of 1018 SNPs (Additional file 3). Most of these variants, however, are in *Brahea* or other *Phoenix* species and are not in any date palm samples studied here. Therefore, selecting only variants among date palm samples (Intra-date palm specific SNPs) identified 37 SNPs in the Chloroplast and 168 in the Mitochondria genomes for a total of 205 intra-date palm SNPs.

**Fig. 1:**
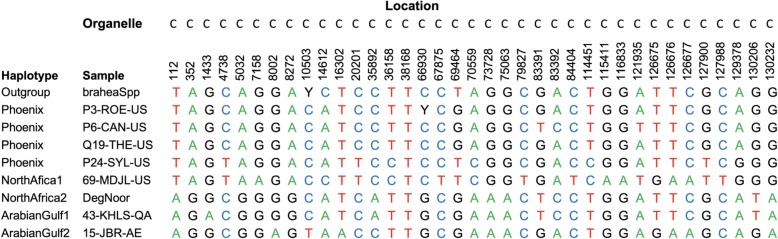
Alleles at intra-date palm specific SNP sites in the Chloroplast. Locations correspond to genome base pair coordinates. Full data set is available in Additional file 2. C: Chloroplast

Among the 205 SNPs that were variable among date palm cultivars, we observed 4 major haplotypes (Fig. 1, Additional file 2). Interestingly, these haplotypes appeared to associate with the origin of the date palm cultivar (Additional file 1). When considering association of haplotypes with geographic origin it is important to note a cultivars historical origin. Commercially important cultivars have now spread across the world such as Medjool that is originally from North Africa yet grown in multiple countries including Jordan, Saudi Arabia and the United States. We noted that, as expected there were two major haplotypes with numerous samples that associated with collection in North Africa (NA1) or the Arabian Gulf (AG1) regions. However, we also detected additional haplotypes in North Africa (NA2) and the Arabian Gulf (AG2), though fewer samples had these haplotypes compared to NA1 and AG1 (Table 2). Of interest was that neither of the regions secondary haplotypes were limited to a single country. Indeed, for North Africa we detected the NA2 haplotype in cultivars originating from Tunisia, Algeria and Egypt. The AG2 haplotype was found in cultivars originating from Iraq, Iran and Oman. Moreover, the AG2 haplotype was more diverged from the AG1 haplotype than was the AG1 from the NA2 (Tables 3 and 4). Indeed, the higher similarity between the NA2 and AG1 haplotypes suggest that the separation of the two groups occurred long after the other 3 (NA1, AG1 and AG2) haplotypes were cultivated. In summary SNP differences between the haplotypes when combining chloroplast and mitochondria (205 total SNPs considered) were as follows: AG1:AG2 96 SNPs, AG1:NA1 158 SNPs, AG1:NA2 10 SNPs, NA1:AG2 146 SNPs, NA1:NA2 156 SNPs, AG2:NA2 96 SNPs (see Tables 3 and 4 for haplotype similarity rather than divergence).

**Table 2:** Haplotype counts among samples in this project

Haplotype	Count
North Africa 1 (NA1)	76*
North Africa 2 (NA2)	18
Arabian Gulf 1 (AG1)	90
Arabian Gulf 2 (AG2)	17

*We found 7 samples within the NA1 haplotype that contained a single base difference and most derived from the “Thoory” cultivar

**Table 3:** Matrix of similarity at Intra-Date Palm SNPs Sites. Only SNPs varying between date palm cultivars are documented, first number is total count of similar SNP calls per total SNPs called between two samples

Mitochondria	AG2	AG1	NA1	NA2	P	P	P	P	O
	15-JBR-AE	43-KHLS-QA	69-MDJL-US	DegNoor	P24-SYL-US	P3-ROE-US	P6-CAN-US	Q19-THE-US	BraheaSpp
Sample									
15-JBR-AE	168/168	83/168	50/168	82/168	79/168	95/160	98/166	100/167	75/130
43-KHLS-QA	83/168	168/168	43/168	159/168	74/168	94/160	89/166	93/167	70/130
69-MDJL-US	50/168	43/168	168/168	44/168	129/168	91/160	108/166	102/167	68/130
DegNoor	82/168	159/168	44/168	168/168	75/168	93/160	88/166	94/167	68/130
P24-SYL-US	79/168	74/168	129/168	75/168	168/168	112/160	119/166	116/167	75/130
P3-ROE-US	95/160	94/160	91/160	93/160	112/160	160/160	128/159	129/160	93/125
P6-CAN-US	98/166	89/166	108/166	88/166	119/166	128/159	166/166	143/165	92/130
Q19-THE-US	100/167	93/167	102/167	94/167	116/167	129/160	143/165	167/167	94/130
BraheaSpp	75/130	70/130	68/130	68/130	75/130	93/125	92/130	94/130	130/130
Chloroplast									
Sample									
15-JBR-AE	37/37	26/37	9/37	27/37	15/37	23/37	20/37	23/37	21/37
43-KHLS-QA	26/37	37/37	4/37	36/37	16/37	24/37	25/37	24/37	21/37
69-MDJL-US	9/37	4/37	37/37	5/37	25/37	16/37	16/37	17/37	18/37
DegNoor	27/37	36/37	5/37	37/37	17/37	25/37	26/37	25/37	22/37
P24-SYL-US	15/37	16/37	25/37	17/37	37/37	28/37	26/37	29/37	28/37
P3-ROE-US	23/37	24/37	16/37	25/37	28/37	37/37	33/37	36/37	33/37
P6-CAN-US	20/37	25/37	16/37	26/37	26/37	33/37	37/37	34/37	31/37
Q19-THE-US	23/37	24/37	17/37	25/37	29/37	36/37	34/37	37/37	34/37
braheaSpp	21/37	21/37	18/37	22/37	28/37	33/37	31/37	34/37	37/37

**Table 4:** Similarity matrix between samples of all SNPs. All documented SNPs are included, first number is total count of similar SNP calls per total SNPs called between two samples

	AG2	AG1	NA1	NA2	P	P	P	P	O
SAMPLE	15-JBR-AE	43-KHLS-QA	69-MDJL-US	DegNoor	P24-SYL-US	P3-ROE-US	P6-CAN-US	Q19-THE-US	braheaSpp
15-JBR-AE	1018/1018	920/1018	871/1018	918/1018	860/1018	491/997	719/1014	784/1016	291/851
43-KHLS-QA	920/1018	1018/1018	859/1018	1004/1018	856/1018	492/997	716/1014	779/1016	286/851
69-MDJL-US	871/1018	859/1018	1018/1018	859/1018	921/1018	481/997	726/1014	781/1016	281/851
DegNoor	918/1018	1004/1018	859/1018	1018/1018	856/1018	492/997	713/1014	778/1016	285/851
P24-SYL-US	860/1018	856/1018	921/1018	856/1018	1018/1018	489/997	739/1014	783/1016	296/851
P3-ROE-US	491/997	492/997	481/997	492/997	489/997	997/997	527/994	524/997	419/836
P6-CAN-US	719/1014	716/1014	726/1014	713/1014	739/1014	527/994	1014/1014	787/1012	319/851
Q19-THE-US	784/1016	779/1016	781/1016	778/1016	783/1016	524/997	787/1012	1016/1016	323/850
braheaSpp	291/851	286/851	281/851	285/851	296/851	419/836	319/851	323/850	851/851

A fifth chloroplast haplotype was noted but only contained a single distinguishing position from the NA1 haplotype at bp 38,168. It was found in the “Thoory” cultivar, its known progeny from crosses (Additional file 1) and some cultivars developed in the USA that are likely derivatives of these crosses. The progeny of these crosses, despite, including paternal males from the Arabian Gulf, confirm that maternal transmission of the chloroplast and mitochondria is the norm in date palm.

**Fig. 2:**
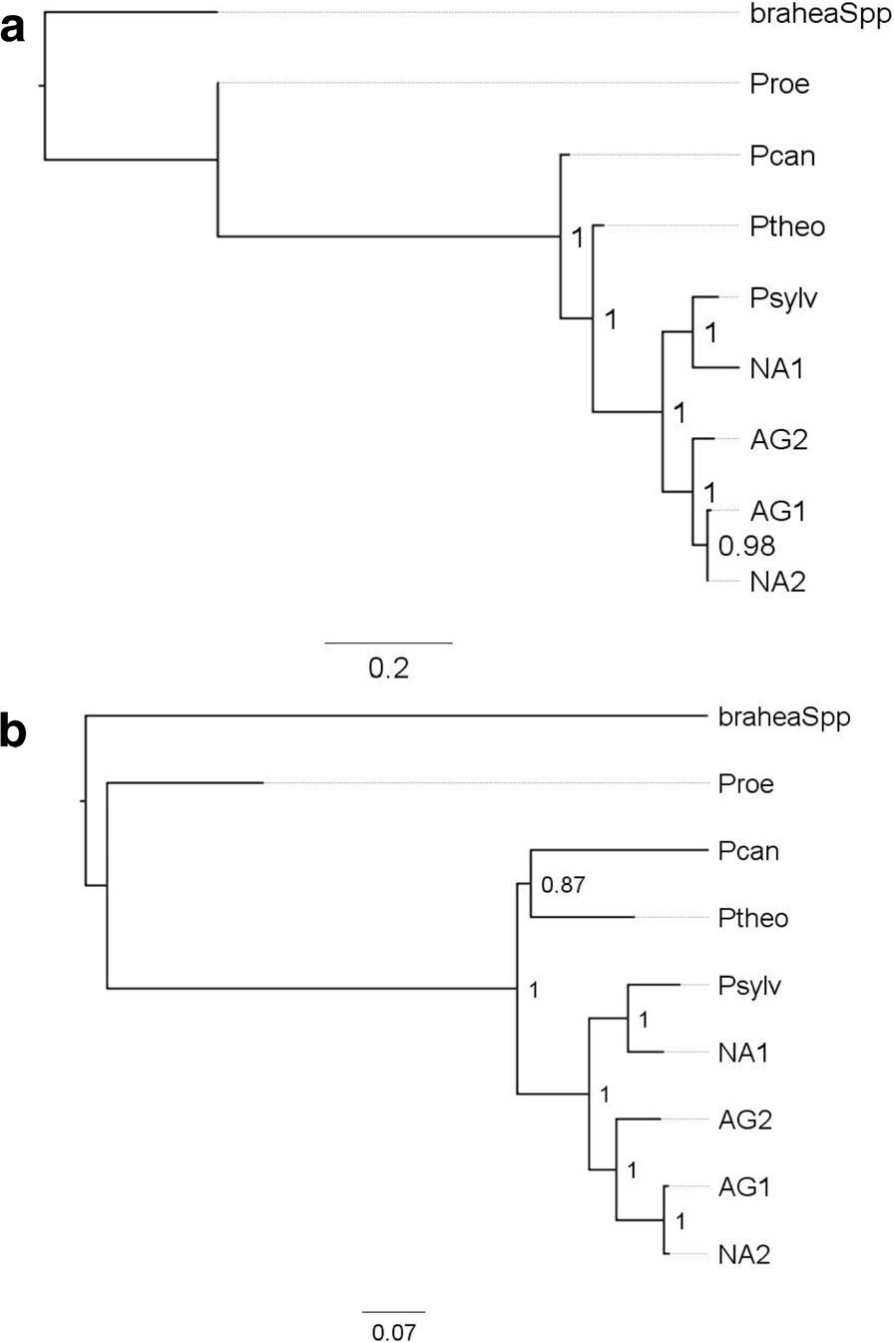
Maximum likelihood based Phylogenetic tree of haplotypes detected in this study. **a** based on Chloroplast SNPs, **b** – based on Mitochondrial SNPs. Numbers at the branch point represent frequency of branching in 100 bootstrapped trees. BraheaSpp: *Brahea dulcis*, Proe: *Phoenix roebelenii*, Pcan: *Phoenix canariensis*, Ptheo: *Phoenix theophrasti*, Psylv: *Phoenix sylvestris*, Haplotype labels are AG1: Arabian Gulf 1, AG2: Arabian Gulf 2, NA1: North Africa 1, NA2: North Africa 2

To better understand the phylogenetic relationship of the organelle haplotypes, SNPs from the chloroplast or mitochondrial were used for phylogenetic tree construction. We selected single representatives from each of the four date palm haplotype groups and included multiple *Phoenix* species for comparison and *Brahea dulcis* as outgroup. Maximum-likelihood phylogenetic analysis revealed that the NA1 haplotype is significantly differentiated from the other haplotypes. An observation that agrees with previous phylogenetic analysis of nuclear markers for the North African cultivars [7, 8]. The NA1 date palm haplotype branched from *P. sylvestris* confirming the close relationship observed by others [3, 6, 19] (Fig. 2).

Other groups studying chloroplast markers from Deglet Noor, a cultivar from Algeria and Tunisia have noted its similarity to Arabian Gulf cultivars [6, 19]. Indeed, the chloroplast of Deglet Noor (NA2) had a single difference to AG1, however, multiple distinguishing differences between the NA2 and AG1 haplotypes were found among mitochondrial markers (Additional file 2) and these were confirmed in cultivars from other countries (Table 1). We never observed mixing of mitochondrial and chloroplast haplotypes in a single cultivar as expected by the almost exclusive maternal transmission of the organellar genomes.

## Discussion

By utilizing organellar genome sequencing we have identified two additional haplotypes representing subpopulations beyond the currently known North Africa/Arabian Gulf separation. While others have observed some further genetic subdivision of nuclear markers within the major populations [20], the sources of these subdivisions were not noted to be related to possible original maternal contributions to cultivar groups. The subpopulations identified here further distinguish cultivar origins within the main regions offering insight into the history of date palm cultivation.

Of interest was the identification of a significantly diverged second Arabian Gulf haplotype (AG2). AG2 is certainly closer to the AG1 haplotype (96/205 SNP differences combining chloroplast and mitochondria) or AG1 related NA2 haplotype (96/205 SNP differences) than the NA1 haplotype (146/205 SNP differences). However, the divergence between AG2 and AG1 is high when compared to the divergence of NA2 and AG1 (10/205 SNP differences) (Tables 3 and 4). This suggests that the AG2 haplotype may represent a third early center of date palm cultivation with a significantly diverged maternal contributor (discussed below). The low number of SNPs between AG1 and NA2 suggests these two separated from each other much later than did the second Arabian Gulf haplotype (AG2). Likewise, the most common North African haplotype (NA1) is highly diverged from the Arabian Gulf haplotypes and likely represents a distinct, early center of date palm cultivation. Altogether, the genetic distinction among the 3 major haplotypes (AG1, AG2, NA1) suggests their geographic separation at the time of initial cultivation. That is, the haplotypes are highly diverged from each other so were unlikely to have been first cultivated in the same region and at the same time.

The similarity of the major North African haplotype to *P. sylvestris* is important to note and agrees with the findings of Flowers and colleagues in their analyses of the date palm chloroplast and mitochondrial genomes [4]. Their findings show that while introgression from *P. theophrasti* occurred in the cultivation of the North African date palm, this was likely through male contribution as the chloroplast and mitochondrial genomes retain their close relationship to *P. sylvestris*. *P. sylvestris* is native to South Asia [21] and so closer to the regions cultivating the AG1 and AG2 haplotypes. It is possible that the maternal contributor to the major North African haplotype was *P. sylvestris* but how this would occur geographically requires further investigation. Nuclear markers from cultivars in this region show distinction from Arabian Gulf cultivars and are at the base of the date palm phylogenetic tree closer to other *Phoenix* species [7].

Whether the combination of nuclear and organellar information is indication of a highly distinct, ancient date palm in North Africa or simply introgression with *P. sylvestris* will require further research.

While the use of nuclear DNA markers assists in understanding admixture of populations, organellar genome markers can assist in understanding more simple maternal contributions. We see concordance with previous results from across the date palm cultivating regions that genotyped specific chloroplast markers and found 2 major haplotypes in date palm [6]. From the detail offered by genome sequencing, we can extend this to 4 haplotypes. Our results on the presence of a second chloroplast and mitochondrial haplotype in the Arabian Gulf agree with Flowers and colleagues [4], however in contrast, we see distinction between two North African chloroplast and mitochondrial haplotypes. These finding agree with both Zehdi-Azouzi and colleagues [6] and Pintaud and colleagues [19] that the chloroplast haplotype found in the group including Deglet Noor is genetically closer to the Arabian Gulf haplotype than to the major North African haplotype. Our results stand in contrast to others who have utilized just portions of the date palm chloroplast genome for sequence analysis. In Tunisian cultivars, some groups have found 8 haplotypes among 12 samples [16] or 14 haplotypes among 31 samples [15] utilizing the *trnL* intron or *trnL-trnF* spacer. Likewise 5 haplotypes were found in 30 Emirati cultivars [18] and 3 major groups in 8 Saudi Arabian cultivars [17]. These groups used PCR amplification followed by Sanger Sequencing and included insertions and deletions in their analysis but the discrepancy between the number of distinct haplotypes we observed is clear. We do not believe this is a result of false-negative SNP calls in the variable regions as we are able to call SNPs in these regions from Phoenix species or the outgroup palm. It is possibly a limitation of the stringency of SNP calling we utilized to ensure low false-positive SNP calls and that loosening these would identify additional minor subgroups within the major 4 haplotypes as occurred with the ‘Thoory’ derived cultivars. Nevertheless, it is clear that we only observe 4 major chloroplast and mitochondrial haplotype groups across the date palm growing world. We may identify additional ones in the future but these 4 haplotypes include a majority of the most famous and commercially important cultivars.

Based on the observation that the NA2 haplotype is more similar to the AG1 than any other haplotype suggests two possibilities. A recent ancestor of the NA2 haplotype may have been a maternal contributor to the AG1 cultivars or vice-versa. We propose that it was likely the NA2 haplotype that derived from the AG1 as they are both closer to the other Arabian Gulf haplotype (AG2) than the major North African 1 haplotype. This would then suggest that there were 3 major centers of date palm cultivation, two in the Arabian Gulf and one in North Africa. A fourth that derived from one of the Arabian Gulf cultivars then spread and includes the famous North African “Deglet Noor” and Egyptian “Zaghlool” cultivars.

The fact that we did not observe mixing of the haplotypes in all the cultivars studied here suggests that the haplotypes came into existence prior to the spread of cultivars and that transmission of the mitochondria and chloroplast is indeed tightly linked. Whether the centers of cultivation were initiated by transfer of male contributors from other regions, as was observed in the major North African cultivars or rather contribution occurred later in the cultivation process remains to be studied for the second Arabian Gulf haplotype. However, it is clear that the female contribution to each center was unique based on the haplotypes observed here.

## Conclusions

The strong distinction between the haplotypes found here argues against a single center of date palm cultivation whose cultivars then spread to other regions with a bottleneck creating significant distinction. Rather, it suggests that there were likely 3 distinct centers of cultivation from which cultivars in those regions all derived from a single maternal contributor followed by a fourth that developed from the AG1 haplotype. These centers of cultivation were then responsible for hundreds of future cultivars that are now available with admixture of the nuclear genome occurring at the boundaries of these centers. The proximity of the most common North African haplotype to *P. sylvestris* requires further investigation and may explain some of the previously observed genetic structure in the overall date palm population. Altogether, these results inform our understanding of the earliest origins of date palm cultivation.

## Methods

### Sample collection and genome sequencing

Date fruit samples were from the Qatar date fruit biobank [22], a collection of date fruit samples from across the date palm growing region spanning from Morocco in the West to Pakistan in the East (Additional file 1). Briefly, the fruit samples in the Qatar date fruit biobank were obtained from commercial outlets in the country of collection or local farms with identification by the product packaging or farmer. We attempted to select the most important commercial cultivars as well as lesser known varieties so as to represent the genetic diversity in regions. We also sequenced a subset of *Phoenix* species identified by and collected from the USDA palm collection and the outgroup palm *Brahea dulcis* identified by and collected from the Huntington library botanical garden palm collection (San Marino, CA, USA). DNA from fruit for date palm, or leaves for other species, was extracted as described [7]. Sequencing libraries were constructed from total DNA and sequenced on Illumina HiSeq 2500/4000 instruments with paired 150 bp reads according to the manufacturers recommended protocol.

### Sequence analysis

Sequences were aligned to the complete date palm chloroplast (NCBI ID NC_013991.2, GI:300399125) and mitochondrial (NCBI ID NC_016740.1, GI:372450205) reference genomes of the Eastern cultivar Khalas [23, 24] using BOWTIE2 [25] and Single Nucleotide Polymorphisms (SNPs) called with SAMTOOLS [26]. We removed sites that were heterozygous in multiple date palms as these are likely duplicated, repetitive or nuclear transferred mitochondrial (NucMt) sequences rather than simple sequence errors or heteroplasmy. In one analysis, a single alternative allele was required in at least one of the date palms analyzed for a SNP to be called across the population (Intra-date palm specific SNPs). A second analysis simply required a variant in any sample including other *Phoenix* and outgroup palms. We excluded insertions or deletions and required an overall population SNP call quality of greater than 900.

### Phylogenetic analysis

Polymorphic sites in the form of a VCF file were transformed into PHYLIP formatted sequence using VCF2PHYLIP [27]. We conducted phylogenetic analysis with PhyML [28] using both bootstrap and ML approaches. Phylogenetic trees were plotted with FIGTREE (http://tree.bio.ed.ac.uk/software/figtree/).

## Additional files

Additional file 1: Cultivar Information. Table containing information on date palm cultivars analyzed in this study. (XLSX 17 kb)

Additional file 2: Mitochondrial and Chloroplast Haplotype SNP Positions. Table containing genotypes for all intra-date palm SNP positions in the Mitochondrial and Chloroplast haplotypes identified in this study. (XLSX 18 kb)

Additional file 3: Date Palm Genotypes. File containing all genotypes utilized in this analysis in vcf format. (VCF 3902 kb)

## Data Availability

All data generated or analysed during this study are included in this published article and its supplementary information files.

## Abbreviations

AG1: Arabian Gulf Haplotype 1 (Chloroplast and Mitochondria)
AG2: Arabian Gulf Haplotype 1 (Chloroplast and Mitochondria)
ML: Maximum likelihood
NA1: North Africa Haplotype 1 (Chloroplast and Mitochondria)
NA2: North Africa Haplotype 2 (Chloroplast and Mitochondria)
NCBI: National Center for Biotechnology Information
NucMt: Nuclear transferred mitochondrial sequences
SNP: Single Nucleotide Polymorphism
USDA: United States Department of Agriculture
